# 24 h Holter Monitoring and 14-Day Intermittent Patient-Activated Heart Rhythm Recording to Detect Arrhythmias in Symptomatic Patients After Severe COVID-19—A Prospective Observation

**DOI:** 10.3390/jcm14082649

**Published:** 2025-04-12

**Authors:** Andrzej Kułach, Michał Kucio, Michał Majewski, Zbigniew Gąsior, Grzegorz Smolka

**Affiliations:** 1 Department of Cardiology, SHS, Medical University of Silesia, Ziolowa 47, 40-635 Katowice, Poland; 2 2nd Division of Cardiology, Upper-Silesian Medical Center, 40-752 Katowice, Poland

**Keywords:** arrhythmia, COVID-19, post-COVID syndrome, palpitations

## Abstract

**Background/Objectives:** COVID-19 is associated with various arrhythmias that continue into a post-COVID period and become a concern for patients and healthcare a long time after the infection. This study aimed to assess the incidence of arrhythmias and their relationship to presented symptoms in patients with no history of rhythm disturbances who underwent severe COVID-19 within the past 6 months. **Methods:** A total of 54 severe COVID-19 survivors with no history of known arrhythmia were enrolled in the study 3–6 months after discharge. All subjects underwent echocardiography, 24 h Holter monitoring, and received a handheld ECG event recorder for 14 days of ambulatory single-lead ECG recording, which was evaluated for supraventricular and ventricular arrhythmias and patient-reported events. After 12 months of follow-up (FU), Holter monitoring and ECG recordings were repeated. **Results:** The incidence of palpitations was high at baseline and halved after 12 months (65% vs. 36%, *p* = 0.018), as was the symptom-induced utilization of the event monitor (36% vs. 12%, p0.012). Palpitations were more common in patients with CAD, diabetes, and hypertension, but were not related to any rhythm disturbances except sinus tachycardia (OR of 5.8 for each 10 bpm increase in HR; CI: 1.3–26.5, *p* = 0.02). Holter monitoring revealed a higher burden of PVCs 3–6 months after COVID vs. FU (PVCs > 200/d in 36% vs. 17%, *p* < 0.05), and PVCs were more commonly recorded events in symptomatic patients. Symptomatic subjects more frequently reported sinus tachycardia (48% vs. 13%, *p* < 0.05) and PVC (21% vs. 0%, *p* < 0.05). Neither arrhythmias nor palpitations were related to the severity of the infection. **Conclusions:** Palpitations are common after severe COVID-19, but the symptoms are related to sinus tachycardia rather than actual arrhythmia and are more pronounced in patients with cardiovascular conditions. Ventricular ectopy was the predominant finding early after severe COVID-19 and might have been responsible for symptoms in a fraction of symptomatic subjects. Both symptoms and sinus tachycardia resolved over time.

## 1. Introduction

The most severe cardiovascular complications of COVID-19 are related to the increased risk of thrombotic events, but the spectrum of SARS-CoV’s effects on the cardiovascular system is wider. Possible mechanisms by which the virus affects the heart include direct damage to the myocardium, cytokine release, systemic inflammation, hypoxia, electrolyte imbalance, and side effects of pharmacological treatment [1]. Therefore, the effect of SARS-CoV-2 infection on the cardiovascular system is much broader and clinically present as myocarditis, heart failure, and arrhythmias [2,3]. In acute settings of the most severe cases of COVID-19, both supraventricular and life-threatening ventricular arrhythmias are not uncommon. They have been reported to be related to worse prognosis and require specific treatment [4,5,6]. The risk of significant arrhythmias in the acute phase is associated with the severity of the COVID-19 disease and the preexisting cardiovascular risk of the patient [7]. Less is known, however, about the prevalence of symptomatic and asymptomatic arrhythmia in convalescents, the types of arrhythmias, their relationship to COVID-19 severity, further prognosis, how long the symptoms and rhythm disturbances persist, and whether they are related. Recent studies have shown that patients with asymptomatic arrhythmias (particularly atrial fibrillation) have the same risk as symptomatic ones and, due to difficulties in diagnosis, even greater risk of progression to permanent arrhythmia [8]. Therefore, systematic rhythm screening, even in asymptomatic subjects, appears justified, particularly in populations at higher risk.

COVID-19 survivors exhibit a wide range of symptoms, likely stemming from diverse mechanisms and multiple body systems involved. Post-COVID syndrome commonly features fatigue, breathlessness, muscle weakness, and palpitations—frequent concerns for patients following SARS-CoV-2 infection [9]. Although palpitations are a prevalent issue, the relationship between these symptoms and specific heart rhythm disturbances remains unclear. In clinical practice, this situation is typically managed through standard or prolonged Holter monitoring [10]. However, this is not an optimal tool for subjects who report rare, short-lasting, and more elusive symptoms.

Patient-activated, handheld, single-lead ECG event recorders have been proposed as useful tools in symptomatic patients (dizziness, palpitations) and, in some clinical settings, are more effective than conventional 24 h Holter–ECG monitoring [11]. Due to their low cost and good specificity in arrhythmia detection, they are being used in many conditions where a proper diagnosis is crucial for further treatment.

This study aimed to evaluate and assess the incidence of various arrhythmias and their relationship to presented symptoms in patients with no history of clinically overt rhythm disturbances who underwent severe COVID-19 within the past 6 months.

Additionally, the patients were followed up for one year to address the persistence of the symptoms and rhythm disturbances.

## 2. Patients and Methods

### 2.1. Screening

We screened 102 consecutive COVID-19 cases hospitalized in a high-volume COVID-19 unit from January to March 2022. The patients were screened during regular cardiology clinic visits offered to patients discharged from the COVID unit in our center, during which they underwent a physical examination, ECG, and echocardiography. The inclusion criteria were severe COVID-19 requiring hospitalization and written consent to participate in the study. We excluded patients with known arrhythmia (including atrial fibrillation and frequent supraventricular and ventricular arrhythmias requiring medication), as well as patients with heart failure, either confirmed by echocardiography or previously diagnosed.

### 2.2. Enrolment

A total of 54 patients were enrolled in this study, 3–6 months (median of 98 days, 95% CI: 94–101 days) after discharge from the COVID-19 unit. All patients consented to participate in the study.

Details on the severity of COVID-19 and the treatment used were retrieved from hospital records and included the following: minimal oxygen saturation, lung damage in CT, oxygen therapy applied (including passive oxygen therapy, high flow, non-invasive ventilation, mechanical ventilation), inflammatory parameters (CRP, interleukin 6, WBC count), other lab parameters (creatinine, troponin T), antiviral/antimicrobial treatment (tocilizumab, remdesivir, steroid, azithromycin), embolic complications (DVT/pulmonary embolism), and duration of hospitalization.

The baseline clinical data included age, sex, BMI, history of diabetes, hypertension, coronary artery disease (proven in angiography, CT-angiography, or with a history of percutaneous intervention or by-pass grafting), COPD (confirmed by spirometry or treated), tobacco smoking, and history of deep vein thrombosis/pulmonary embolism.

A full baseline transthoracic echocardiography was performed, and the following parameters were recorded for study purposes: left ventricle ejection fraction (LVEF), mitral regurgitation, left atrium diameter (LA), and volume (LAV), and right ventricle diameter (RVOT). Patients with EF < 50% or severe valvular disease were excluded.

### 2.3. Holter and Event Recorder Monitoring

The patients underwent 24 h Holter monitoring (Lifecard CF/Pathfinder SL, Reynolds Medical, Snoqualmie, WA, USA) and received a handheld ECG event recorder (CheckMe Pro, Viatom, Shenzhen, China) for 14 ± 2 days of ambulatory single-lead ECG recording (30 s measurements twice a day plus whenever symptomatic).

In the 24 h Holter monitoring, we recorded the maximal, minimal, and average heart rates, the number of premature atrial (PACs) and ventricular complexes (PVCs), episodes of supraventricular and ventricular tachycardia, atrial fibrillation, and bradycardia (<50 bpm) and AV conduction disorders. The cutoff point for the significant burden of PACs and PVCs was set to 200/day, as reported in the literature [12,13].

The handheld event recorder strips were analyzed manually by a cardiologist. The maximal HR from the patient’s strip set was recorded, as well as the PVCs, PACs, and supraventricular and ventricular arrhythmias. Patients with at least one strip with particular arrhythmia (PAC/PVC) were recorded as positive for this arrhythmia. The burden was not analyzed. Additionally, the number of symptom-induced recordings was noted, along with the corresponding findings.

After 12 months (median of 12 months, 95% CI: 12–13 months), the subjects were queried for symptoms (palpitations), and 24 h Holter and 14-day ECG event monitoring were repeated, with the same parameters recorded.

### 2.4. Statistical Analysis

Statistical analysis was performed with MedCalc Statistical Software version 22.026 (MedCalc Software Ltd., Ostend, Belgium). The categorical variables are reported as counts and percentages. The continuous variables are presented as the mean ± SD (standard deviation) for normal distribution and the median with a 95% CI (confidence interval) for non-normal distribution. Normality was tested with the Shapiro–Wilk test. In cases of a normal distribution, Student’s *t*-test was performed; otherwise, the Wilcoxon test for paired samples was used. Qualitative parameters were compared using Pearson’s chi-square and McNemar’s test. The Spearman test was used for correlation analysis.

Logistic regression analysis was performed to determine the factors related to palpitations and recorded arrhythmias.

The universal *p*-value level < 0.05 was regarded as statistically significant throughout the analyses.

This investigation was carried out in accordance with the principles outlined in the Declaration of Helsinki.

## 3. Results

The baseline characteristics of the studied group are presented in Table 1. The study group was 40–75 years old (median of 58.5), with significant overweight/obesity (median BMI of 30.8), and a significant proportion had cardiovascular risk factors, predominantly hypertension and tobacco smoking.

The median hospitalization time was 9 days (95% CI: 8–11.5 days). The median percentage of lung parenchyma affected was 50%, and half of the patients required at least high-flow oxygen therapy. The median saturation upon admission was 86%. Details on the inflammatory parameters and the treatments used are shown in Table 1. All patients had their troponin levels assessed, and in 37%, they were over the upper limit of the norm, although the median value was low.

The echocardiographic parameters at the beginning of the study are presented in Table 2. The patients did not differ with regard to age or echocardiographic parameters (ejection fraction, significant valvular disease, and particularly left atrium parameters).

### 3.1. Symptoms and Arrhythmic Findings in Holter and Event Monitor

Table 3 presents the symptoms and arrhythmic findings at the baseline and their changes over time. The proportion of patients experiencing palpitations significantly decreased from 64.8% at baseline to 36.4% after 12 months. At the same time, the average and maximal heart rates (HRs) and supraventricular arrhythmia burden did not change significantly. The PVC burden (expressed as the number of patients with PVCs > 200/d) dropped, as assessed in Holter monitoring. There were no significant differences in other arrhythmia burdens, although AF and nsVT (non-sustained ventricular tachycardia) tended to be more frequent at baseline.

Symptom-induced utilization of patient monitors (number of symptom-triggered recordings) also dropped after one year, while the number of recordings with arrhythmias and sinus tachycardia did not change significantly. Patient-activated handheld ECG recorders were used properly with no apparent operational issues. 97% of ECG strips were of good quality. All records were reviewed manually by a cardiologist.

### 3.2. Palpitations and Arrhythmia

To analyze the relationship between palpitations and arrhythmia, as well as between other findings and clinical variables, we compared patients who declared palpitation at baseline with those who did not. The results are presented in Table 4.

Patients who reported palpitations during the survey were more often hypertensive, diabetic, and suffered from CAD. The presence of palpitations was not related to the severity of COVID-19 (expressed as inflammatory parameters and lung CT results). Moreover, there were no differences in PVC and PAC burden in Holter monitoring between the symptomatic and asymptomatic subjects.

The event monitor strips, however, revealed that the symptomatic patients had a higher HR when the measurement was taken, the incidence of sinus tachycardia was more frequent, and PVCs were also more frequent. When only symptom-driven events were analyzed, out of the 36% of patients who recorded symptoms-triggered strips, 18% had sinus tachycardia, 6% had PVCs, and in 12%, no specific ECG findings were noted.

We performed a logistic regression analysis to identify the independent parameters related to palpitations. We included age, sex, history of CAD, HTN, myocardial damage (troponin), % lungs affected, PAC and PVC burden, HR max in Holter monitoring, and HR max in the event recorder. The analysis showed only the event recorder HR max to be related to palpitations (*p* = 0.02), with an OR of 5.8 (95% CI 1.3–26.5) for each 10 bpm increase in HR max.

We also assessed the differences between the patients with and without any significant arrhythmia in Holter monitoring (defined as PVC or PAC burden > 200/d or AF or nsVT), but there were no differences regarding COVID-19 severity (CRP of 78 vs. 133, *p* = 0.34; WBC of 8.3 vs. 10.6, *p* = 0.09; CT (% lungs) of 46 vs. 52%, *p* = 0.92).

There was no strong correlation between heart rate (as assessed in Holter or event monitoring) and the markers of severity of COVID-19, except that the percentage of lungs affected was related to the HR max during monitoring (R = 0.44; *p* = 0.005). Also, the HR max was negatively correlated with age (r = −0.64, *p* < 0.0001). There was also a moderate correlation between baseline troponin, reflecting myocardial damage, and the severity of COVID, as assessed by CT lung involvement and the white blood count (Table 5).

## 4. Discussion

Severe COVID-19 may cause sustained and potentially life-threatening arrhythmias during the acute phase, which may be linked to myocarditis [2], as well as a systemic inflammatory response and any underlying cardiovascular conditions [14]. The most frequently reported significant acute-phase arrhythmias are supraventricular types (including atrial fibrillation), which often continue into the early post-discharge period [15].

The persistence of clinically significant arrhythmias in an extended follow-up of the COVID-19 convalescents is less commonly seen. Our study shows that the early post-discharge arrhythmias are mostly PVCs, and they become less frequent over time. The presence of ventricular ectopy is likely to be related to subclinical myocardial damage, which was evidenced by speckle-tracking echocardiography in COVID-19 survivors with preserved LV systolic function [16]. In our study, the burden of PVCs was not shown to be related to the severity of COVID-19.

The most common finding in COVID-19 convalescents is sinus tachycardia, a frequent part of post-COVID syndrome. It was found to be related to the severity of COVID-19, vaccination status, CV comorbidities, and a higher rate of healthcare resource utilization [17]. In our study, the maximal HR was also related to the severity of infection (expressed as the percentage of lungs affected in CT), and it was the main finding in patients who reported palpitations (48% of the events recorded for palpitation were sinus tachycardia).

Palpitations, along with breathlessness and fatigue, are the key symptoms in a post-COVID scenario. They are a major concern for patients and a reason for referral to a cardiologist after COVID-19.

Our study shows that palpitations are barely related to actual arrhythmia and are most likely a feeling of perceptible heartbeats during sinus tachycardia. There is, however, a proportion of patients where it is related to PVCs.

Palpitations were more commonly reported by patients with a diagnosed cardiovascular condition (CAD, hypertension). One possible reason is that patients with known conditions are more sensitive to changes in their cardiac symptoms. Another possibility is that in the COVID era, adherence to therapy (i.e., CV medications, including beta-blockers) was lower. This was, however, not evaluated in this study. Finally, palpitations, even though they were frequent as late as 6 months after infection, resolved over time and were less frequent a year after.

Although in most COVID-19 survivors, palpitations seem to be a self-limiting and rather benign symptom, the strategy to prove the underlying rhythm disturbance (or lack of it) seems reasonable for two reasons. First, it allows practitioners to reassure patients with sinus tachycardia or ectopy that the condition is benign, likely to be transient, and may only require monitoring or a short-lasting treatment. Second, it allows for identifying the small proportion of subjects (i.e., atrial fibrillation, high burden of ectopy) in whom further work-up and treatment are required.

### Limitation

The number of patients is a significant limitation of this study. Holter monitoring utilizing a longer recording would also be more informative.

To isolate the effect of COVID-19 on arrhythmias, we decided to exclude patients with heart failure and previously known arrhythmias. This allowed for a more homogenous study group, but it limits the generalizability of the results, particularly in the group most prone to significant arrhythmias.

## 5. Conclusions

Following severe COVID-19, palpitations are a common finding in patients, but the symptoms are related to sinus tachycardia rather than arrhythmia. The symptoms are more pronounced in patients with preexisting cardiovascular conditions, and they become less frequent over time.

The most common types of arrhythmias in COVID-19 survivors are PVCs, which are more pronounced early after the infection and might be responsible for symptoms in a fraction of symptomatic subjects.

Neither arrhythmias nor palpitations are significantly related to the severity of the infection.

Patient-activated handheld event recorders are a good diagnostic option to identify the cause of symptoms in patients after COVID who are concerned about their cardiac symptoms.

## Figures and Tables

**Table 1:** Baseline characteristics of the studied group, including demographics, medical history, and parameters related to COVID-19 severity.

**Parameter**	
Age, years, median (min–max)	58.5 (40–75)
Female gender, *n* (*n*%)	24 (44%)
BMI, median (95% CI)	30.8 (28.6–31.7)
**Medical history**	
Hypertension, *n* (*n*%)	31 (58.5%)
Diabetes, *n* (*n*%)	8 (15.4%)
Coronary artery disease, *n* (*n*%)	9 (17.3%)
Tobacco smoking, *n* (*n*%)	22 (40.7%)
COPD *n* (*n*%)	0 (0%)
DVT/pulmonary embolism *n* (*n*%)	2 (3.8%)
**COVID-19 hospitalization**	
Hospitalization duration, days, median (95% CI)	9 (8–11.5)
Oxygen saturation on admission, median (95% CI)	86 (82–91)
CRP, mg/L, median (95% CI)	79 (72–134)
Interleukin-6, pg/mL, median (95% CI)	22 (17–34)
WBC (×10^6^/mL), median (95% CI)	11 (8–11.3)
% lungs affected in CT, median (95% CI)	50% (39–55%)
Troponin T, ng/mL, median (95% CI)	0.012 (0.009–0.015)
Troponin T exceeding ULN (>0.014 ng/mL)	17 (37%)
Oxygen therapy type (passive oxygen/high flow/NIV/mechanical ventilation), *n*%	55.6%/24.1%/14.8%/5.6%
Remdesivir treatment, *n* (*n*%)	11 (24.4%)
Tocilizumab treatment, *n* (*n*%)	8 (17%)
Steroid treatment, *n* (*n*%)	34 (73.9%)
Antibiotic (azithromycin), *n* (*n*%)	15 (32.6%)

BMI, body mass index; CI, confidence interval; NIV, non-invasive ventilation; ULN, upper limit of norm; WBC, white blood cell; COPD, chronic obstructive pulmonary disease; DVT, deep vein thrombosis.

**Table 2:** Echocardiographic parameters at time of inclusion to the study.

LVEF, %, median (95% CI)	60 (55–60)
LAV, mL, median (95% CI)	61 (55–73)
Mitral regurgitation (none/mild/moderate/severe), *n*%	20%/66%/12%/0%
RVOT, mm, median (95% CI)	31 (28–31)

LVEF, left ventricle ejection fraction; LAV, left atrium volume; RVOT, right ventricle outflow tract.

**Table 3:** Baseline and follow-up data regarding palpitations, Holter monitoring, and patient monitor findings.

*n* (*n*%)	Baseline (*n* = 54)	After 12 Months (*n* = 54)	*p* Value
**Palpitations**	**35 (64.8%)**	**16 (36.4%)**	**0.0018**
**24 h Holter**			
HR max, bpm, median (95% CI)	108 (106–112)	101 (99–108)	NS
HR avg, bpm, median (95% CI)	68 (64–72)	64 (61–70)	NS
PACs > 200/day	8 (18.2%)	9 (25%)	NS
nsSVT/SVT/AF	6 (13.6%)	3 (7%)	NS
*PVCs > 200/day*	*16 (36%)*	*7 (17%)*	*0.039*
nsVT	2 (4%)	0	NS
AV conduction abnormalities (>1st degree AV block)	0	0	NS
**Patient Monitor**			
No. of recordings (95% CI)	30 (28–34)	26 (24–30)	NS
HR max, bpm, median (95% CI)	93 (88–100)	84 (81–99)	NS
*Symptom-triggered recording*	*16 (36%)*	*5 (12%)*	*0.012*
Sinus tachycardia	7 (16%)	5 (12.2%)	NS
PACs	6 (14%)	4 (9%)	NS
PVCs	7 (16%)	4 (9%)	NS

HR, heart rate; PAC, premature atrial complex; PVC, premature ventricular complex; SVT, supraventricular arrhythmia; AF, atrial fibrillation; nsVT, non-sustained ventricular tachycardia.

**Table 4:** Differences in clinical parameters and rhythm disturbances between patients who reported palpitations and those who did not.

	No Palpitations	Palpitations	*p* Value
**Age, years, median (95% CI)**	**63 (61–67)**	**57 (55–59)**	**0.0007**
Female gender, *n* (*n*%)	58%	54%	0.801
**Hypertension, *n* (*n*%)**	**33%**	**71%**	**0.008**
**Diabetes, *n* (*n*%)**	**0%**	**22%**	**0.034**
**Coronary artery disease, *n* (*n*%)**	**0%**	**26%**	**0.023**
CRP, mg/L, median (95% CI)	138 (108–70)	98 (71–125)	0.057
Interleukin-6, pg/mL, median (95% CI)	43 (16–71)	25 (19–32)	0.119
% lungs affected in CT, median (95% CI)	56 (45–67)	42 (36–52)	0.083
Troponin T > ULN (0.014 ng/mL)	41%	34%	0.653
Holter PACs > 200/d	27%	15%	0.372
Holter PVCs > 200/d	18%	42%	0.152
**PM sinus tachycardia**	**13%**	**48%**	**0.024**
PM PACs	27%	10%	0.164
**PM PVCs**	**0%**	**21%**	**0.061**
**PM HR max, bpm, median (95% CI)**	**84 (69–91)**	**99 (92–106)**	**0.0004**

CRP, C-reactive protein; CT, computed tomography; PM, patient monitor; HR, heart rate; PAC, premature atrial complex; PVC, premature ventricular complex.

**Table 5:** Correlation between troponin T and heart rate assessed in Holter monitoring and event recorder with parameters of COVID severity.

Variable 1	Variable 2	Spearman R	*p*
Troponin T	CRP	0.21	0.17
Troponin T	WBC	0.51	<0.001
Troponin T	CT—% lungs affected	0.55	<0.001
Holter HR average	CRP	0.32	0.04
Holter HR average	CT—% lungs affected	0.35	0.03
HR max (monitor)	CRP	−0.29	0.07
HR max (monitor)	CT—% lungs affected	0.44	<0.001
HR max (monitor)	Age	R = −0.64	<0.001

CRP, C-reactive protein; CT, computed tomography; WBC, white blood cell count.

## Data Availability

The original contributions presented in this study are included in the article. Further inquiries can be directed to the corresponding author.
